# High-resolution diffusion MRI at 7T using a three-dimensional multi-slab acquisition

**DOI:** 10.1016/j.neuroimage.2016.08.054

**Published:** 2016-12

**Authors:** Wenchuan Wu, Benedikt A. Poser, Gwenaëlle Douaud, Robert Frost, Myung-Ho In, Oliver Speck, Peter J. Koopmans, Karla L. Miller

**Affiliations:** a FMRIB Centre, Nuffield Department of Clinical Neurosciences, University of Oxford, Oxford, UK; b Department of Cognitive Neuroscience, Maastricht Brain Imaging Centre, Faculty of Psychology & Neuroscience, Maastricht University, PO Box 616, 6200MD Maastricht, The Netherlands; c Department of Neurologic Surgery, Mayo Clinic, Rochester, MN, USA; d Department of Biomedical Magnetic Resonance, Otto-von-Guericke-University Magdeburg, Magdeburg, Germany; e Leibniz Institute for Neurobiology, Magdeburg, Germany; f Center for Behavioral Brain Sciences, Magdeburg, Germany; g German Center for Neurodegenerative Disease, Site Magdeburg, Germany

**Keywords:** Diffusion, High resolution, 7T, Tractography, 3D, Multi-slab

## Abstract

High-resolution diffusion MRI can provide the ability to resolve small brain structures, enabling investigations of detailed white matter architecture. A major challenge for in vivo high-resolution diffusion MRI is the low signal-to-noise ratio. In this work, we combine two highly compatible methods, ultra-high field and three-dimensional multi-slab acquisition to improve the SNR of high-resolution diffusion MRI. As each k_z_ plane is encoded using a single-shot echo planar readout, scan speeds of the proposed technique are similar to the commonly used two-dimensional diffusion MRI. In-plane parallel acceleration is applied to reduce image distortions. To reduce the sensitivity of auto-calibration signal data to subject motion and respiration, several new adaptions of the fast low angle excitation echo-planar technique (FLEET) that are suitable for 3D multi-slab echo planar imaging are proposed and evaluated. A modified reconstruction scheme is proposed for auto-calibration with the most robust method, Slice-FLEET acquisition, to make it compatible with navigator correction of motion induced phase errors. Slab boundary artefacts are corrected using the nonlinear slab profile encoding method recently proposed by our group. In vivo results demonstrate that using 7T and three-dimensional multi-slab acquisition with improved auto-calibration signal acquisition and nonlinear slab boundary artefacts correction, high-quality diffusion MRI data with ~1 mm isotropic resolution can be achieved.

## Highlights

• High-resolution (1 mm) in vivo diffusion MRI with high SNR.
• 3D multi-slab acquisition at 7T for high SNR efficiency.
• Motion-robust parallel imaging kernel for 3D multi-slab acquisition.
• Reducing slab boundary artefacts using nonlinear slab profile encoding.
• High-quality fibre tractography with excellent anatomical fidelity.

## 1 Introduction

Diffusion MRI has been widely used in the investigation of neuroanatomical connections. Although the possibility of in vivo high-resolution diffusion MRI is compelling, the feasibility of this technique in practice is still limited. The primary challenges include motion sensitivity and the reduced SNR at high spatial resolution, in which the latter is particularly problematic for diffusion MRI due to its low intrinsic SNR, which often means imaging near the noise floor. To improve the SNR of in vivo high-resolution diffusion MRI without substantially increasing the scan time, the two dominant approaches are the use of ultra-high field (7T and up) scanners (Heidemann et al., 2012, Eichner et al., 2014, Strotmann et al., 2014, Vu et al., 2015) and acquisition schemes with higher SNR per unit time compared to the current standard, two dimensional (2D) single-shot echo planar imaging (EPI) (Van et al., 2011, Setsompop et al., 2012a, Engstrom and Skare, 2013, Uğurbil et al., 2013, Frost et al., 2014, Chang et al., 2015).

Ultra-high field MRI can provide stronger signals than more standard field strengths (e.g. 3T), which could be used to preserve the SNR at higher spatial resolution. The downside of ultra-high field however is that *T*_2_ and *T*_2_^*^ become shorter, which to some extent counteracts the SNR benefit of high field because of the long echo time (TE) associated with spin-echo diffusion preparation (Van Essen and Ugurbil, 2012, Uğurbil et al., 2013). Short *T*_2_^*^ and strong *B*_0_ inhomogeneity are the causes of two major EPI artefacts, image blurring and distortion, which can dramatically degrade the image quality, particularly at high field. Hence, parallel acceleration (Sodickson and Manning, 1997, Pruessmann et al., 1999, Griswold et al., 2002) becomes crucial for single-shot EPI at 7T to reduce the echo train length (less *T*_2_^*^ blurring) and decrease the effective echo spacing (less distortion) (Vu et al., 2015). Although segmented EPI could serve as an alternative solution for reducing EPI artefacts (Butts et al., 1996, Porter and Heidemann, 2009), the long scan time and practical challenges associated with segmented EPI acquisition due to motion sensitivity and phase errors might hinder its application in many diffusion MRI studies. Another downside of 7T is the increased specific absorption rate (SAR), which is a great challenge for spin-echo diffusion MRI, especially when multi-band acceleration is applied. Even without multi-band acceleration, the increased SAR could still limit the RF pulses (especially the refocusing pulse) that can be used at 7T.

SNR can also be improved by addressing fundamental inefficiencies in the acquisition. Optimal SNR efficiency for spin-echo MRI to depict white matter is associated with TR in the range of 1–2 s (Frost et al., 2014), with longer TR having poor efficiency and shorter TR suffering from signal saturation. In conventional 2D acquisitions the TR is a direct consequence of the number of slices, typically requiring long TR (7–10 s) to achieve full brain coverage for high-resolution acquisitions. Three-dimensional (3D) single-slab acquisitions de-couple the coverage from TR, but are only efficient with very short TR (~100 ms). Hence, neither 2D nor 3D can achieve optimal SNR efficiency. Two classes of hybrid 2D/3D acquisition have emerged in recent years that move closer to the optimally efficient TR. Simultaneous multi-slice imaging uses parallel imaging to remove the strict coupling of 2D slices to TR (Larkman et al., 2001, Moeller et al., 2010, Setsompop et al., 2012b, Setsompop et al., 2013); however, the number of slices that can be acquired simultaneously is limited in practice, and optimal SNR efficiency cannot at present be achieved without sacrificing coverage (particularly at 7T). 3D multi-slab acquisition takes the opposite approach, by covering the whole volume using multiple 3D slabs and defining slices within each slab using *k*-space encoding. The TR of 3D multi-slab acquisition is proportional to the number of slabs, which can be adjusted to favour a TR associated with optimal SNR efficiency, making this a very attractive method for maximising SNR (Engstrom and Skare, 2013, Frost et al., 2014).

The higher signal at 7T and optimal SNR efficiency from 3D multi-slab acquisition are complementary, such that the combination of these two methods should be able to provide high-quality diffusion MRI images. However, there are still two problems to be solved, which could otherwise lead to substantially deterioration of the image quality.

First, parallel imaging methods that require auto-calibration signal (ACS) measurements (Griswold et al., 2002) are greatly affected by the quality of ACS data, particularly for EPI-based acquisitions (Polimeni et al., 2016). Single-shot ACS data have different geometric distortions than the imaging data they are applied to, leading to residual artefacts; conversely, segmented ACS acquisitions match distortions but are sensitive to subject motion and respiration, again leading to artefacts. Recently, it has been shown that a re-ordering of the ACS acquisition scheme (Talagala et al., 2015, Polimeni et al., 2016) can provide more robust ACS data in the presence of motion and respiration as well as proving more robust to strong *B*_0_ offset (Polimeni et al., 2016). In this work, we extend these alternative ACS acquisitions to 3D multi-slab acquisition to improve the robustness and performance of the GRAPPA reconstruction.

Second, 3D multi-slab exhibits slab boundary artefacts because the edges of the slab profiles cannot be perfectly sharp and abutting, resulting in periodic signal modulations due to saturation at the boundaries and slab aliasing (Van et al., 2015, Wu et al., 2015). A number of methods for reducing these artefacts have been proposed (Oshio et al., 1991, Parker et al., 1991, Murakami et al., 1995, Van et al., 2011, Engstrom and Skare, 2013, Frost et al., 2014, Chang et al., 2015), all of which increase scan time due to oversampling/overlapping of slabs, and often require a longer TR to reduce the saturation effects at slab boundaries (Engstrom et al., 2015). The slab profile encoding (PEN) method (Van et al., 2015) can correct the aliasing artefacts with minimal increase in scan time (except for a calibration scan), but exhibits residual artefacts below TR~4 s (Wu et al., 2015). We recently proposed the nonlinear slab profile encoding (NPEN) extension, which corrects both slab aliasing and signal modulations, while being compatible with optimal TR (Wu et al., 2015). The performance of NPEN correction has already been demonstrated at 3T; here, NPEN is applied at 7T to reduce the slab boundary artefacts for 3D multi-slab diffusion MRI.

Here, we leverage the compatibility of 7T and 3D multi-slab acquisition to achieve high-resolution, high-SNR diffusion MRI. We compare different ACS acquisition strategies for 3D multi-slab imaging. Slab boundary artefacts are corrected using the NPEN method, which has required further improvements to handle larger *B*_0_ offsets at 7T. With all these advances, we achieve in vivo diffusion MRI data at 7T with high resolution and high quality. Diffusion analysis and tractography on these data are presented alongside published post-mortem results, demonstrating consistent findings.

## 2 Theory

The fast low angle excitation echo-planar technique (FLEET) is a hybrid of FLASH and segmented-EPI (Chapman et al., 1987, Polimeni et al., 2016). Unlike conventional segmented EPI, which uses an inner slice loop and cycles through segments in the outer loop to favour recovery of longitudinal magnetisation, FLEET swaps the order of slice and segment loops, such that all segments for a given slice are fully acquired before moving to the next slice. This reduces the temporal separation between segments of a single slice, minimizing the effects of subject motion and respiration. These favourable properties of FLEET type segmented acquisition make it highly suitable for the use as ACS module in 2D EPI sequences (Polimeni et al., 2016) and this also extends to 3D EPI (Ivanov et al., 2015).

**Fig. 1:**
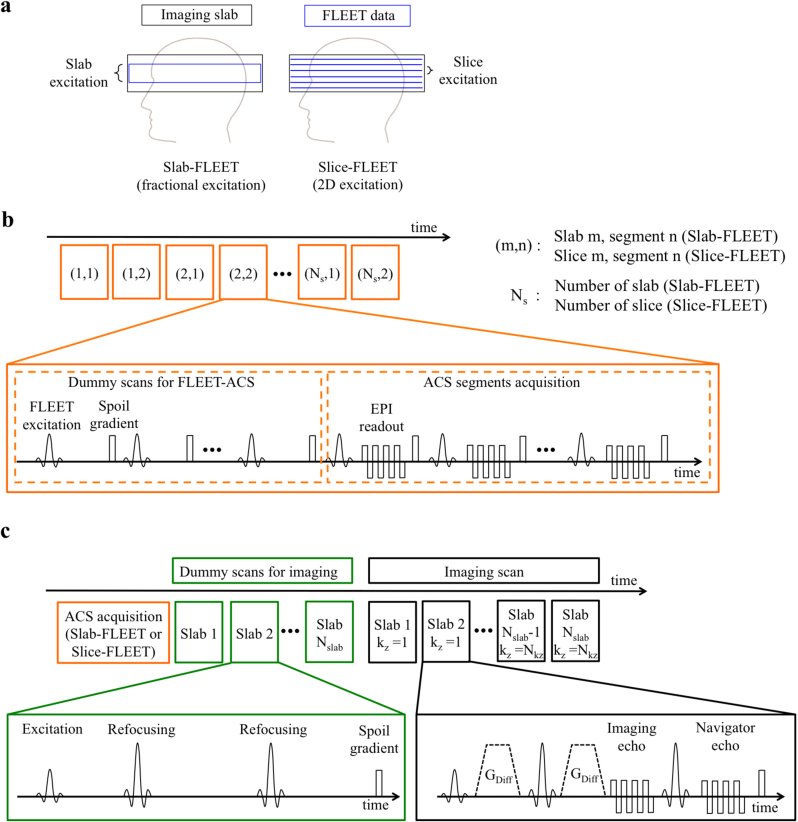
Illustration of 3D multi-slab acquisition with FLEET-ACS. (a) Two possible schemes for implementing FLEET-ACS in 3D multi-slab acquisition. (b) Schematics for FLEET-ACS acquisition, GRAPPA factor of 2 is assumed in this example. Both Slab-FLEET and Slice-FLEET utilize consecutive-segment acquisition order, such that the interval between the acquisition of the first and the last segments for a given slice/slab can be minimised. (c) Schematics for imaging data acquisition, the orange block represents the full FLEET-ACS acquisition shown in (b). *N*_slab_ is the number of slabs. *N*_kz_ is the number of *k*_z_ phase encodes for each slab.

To combine FLEET-ACS and 3D multi-slab acquisition, two possible schemes are immediately apparent (Fig. 1a): ‘Slab-FLEET’, which excites slabs aligned to the centre of the subsequent imaging slab data without *k*_z_ phase encoding, and ‘Slice-FLEET’, which excites 2D slices with locations matching to the final imaging volumes. Slice-FLEET obtains reference data for all slices, whereas Slab-FLEET acquires one set of reference for all slices in a given slab, resulting in a shorter calibration scan. The two schemes are expected to differ in three key aspects: (i) intra-voxel dephasing, (ii) coil profile mismatch, and (iii) acquisition time, which are discussed below:

(i) FLEET is a gradient-echo based sequence, which suffers from signal dephasing inside a voxel, exacerbated by the strong *B*_0_ inhomogeneity at 7T and thick excitations. Because Slab-FLEET excites a thick slab (without k_z_ phase encoding), it is worth considering slab excitations that are thinner than the imaging slab (which is *k*_z_ phase encoded) (Fig. 1a). Slice-FLEET will have considerably less intra-voxel dephasing due to the thin slices.
(ii) Slab-FLEET essentially averages the coil sensitivity of the whole FLEET slab. This strategy assumes the coil sensitivity is relatively constant along the slab direction, but in reality the averaged coil profile is not optimal for GRAPPA reconstruction at each individual slice. If thinner Slab-FLEET excitation is utilised to reduce intra-voxel dephasing, slices at slab boundaries would have more inaccurate GRAPPA kernel calibration. In contrast, Slice-FLEET can provide matched coil profile for each slice, potentially enabling more accurate GRAPPA reconstruction.
(iii) Despite the potential disadvantages in coil profile mismatch and intra-voxel dephasing, Slab-FLEET requires only one set of ACS data per slab. By contrast, Slice-FLEET acquires one set of ACS for each slice, requiring longer acquisition time.

## 3 Methods

### 3.1 Sequence

A 2D spin-echo diffusion MRI sequence was modified to acquire 3D multi-slab data with the Stejskal–Tanner preparation scheme (Stejskal and Tanner, 1965). The sequence used the 90° excitation and 180° refocusing pulses provided by the vendor with a time-bandwidth product of 24 and 5 and durations of 7.68 ms and 10.24 ms, respectively. After acquisition of each *k*-space (“imaging”) segment, a second refocusing pulse was applied to acquire a 2D navigator at lower resolution to correct the phase errors induced by subject bulk motion during diffusion encoding (Miller and Pauly, 2003, Engstrom and Skare, 2013). The imaging data were phase-encoded along *k*_y_ and *k*_z_ to generate a 3D *k*-space, whereas the navigator data was only phase encoded along *k*_y_, forming a 2D *k*-space, in which the signal of the excited slab is collapsed across the slab. Use of a 2D navigator has been demonstrated to provide satisfactory correction of phase errors in thin (up to 30 mm) 3D slabs (Frank et al., 2010, Engstrom and Skare, 2013). As the navigator is not *k*_z_ phase-encoded, Slab-FLEET ACS is a more suitable option for GRAPPA reconstruction than Slice-FLEET ACS.

Each *k*_z_ plane was sampled with single-shot EPI (SSH-EPI), enabling a similar sampling efficiency as conventional 2D SSH-EPI sequences. Parallel imaging acceleration using GRAPPA (Griswold et al., 2002) was applied along the *k*_y_ phase encoding direction. Partial Fourier was used to achieve a short TE. The navigator data was also acquired with GRAPPA accelerated SSH-EPI, but the *k*_y_ dimension of the acquisition matrix was kept to 64 lines to reduce the scan time. No partial Fourier was used in the navigator acquisition.

In ACS acquisition, all segments for a given slab (Slab-FLEET) or slice (Slice-FLEET) were acquired consecutively (Fig. 1b). A constant flip angle was used for excitation. Before ACS acquisition, a series of dummy scans were applied to achieve a steady state magnetization, such that all segments would have equal signal level. RF spoiling and gradient spoiling were applied as described previously (Polimeni et al., 2016). The TE of ACS acquisition was minimised to reduce the acquisition time. Fat saturation was applied before RF excitations to suppress the fat signal, which was not shown in the figure. After ACS acquisition, a series of imaging dummy scans were applied, followed by the 3D multi-slab image acquisition (Fig. 1c).

### 3.2 Data acquisition

Six healthy subjects were scanned on a Siemens 7T scanner using a 32-channel RX Nova coil and a SC72D gradient set with maximum gradient amplitude of 70 mT/m on each axis and maximum slew rate of 200 mT/m/ms. Written informed consent in accordance with local ethics was obtained from each subject before experiments. Three subjects were scanned using an ACS evaluation protocol to evaluate the performance of different ACS acquisition methods. The other three subjects were scanned using a high-resolution diffusion protocol.

#### 3.2.1 ACS evaluation protocol

For each subject, 14 slabs with 10 slices per slab were acquired and neighbouring slabs were overlapped by 20% (2 slices), resulting in 114 slices in the final reconstruction. The slab acquisition was interleaved while *k*_z_ encoding inside each slab was sequential. Each *k*_z_ plane was acquired using SSH-EPI, such that the number of segments is equal to the number of k_z_ encoding in each slab. 51 echoes were collected with an echo spacing of 0.82 ms and a bandwidth of 1442 Hz/pixel. GRAPPA acceleration 3 was applied along *k*_y_ phase encoding, resulting in an effective echo spacing of 0.27 ms. Partial Fourier acquisition of 6/8 was used, resulting in a final in-plane matrix of 204×204. The TEs for the EPI readouts were 71 ms (imaging) and 141 ms (navigator); TR was 2600 ms. Isotropic resolution of 1.03×1.03×1.03 mm^3^ was acquired with a FOV of 210×210×117 mm^3^. Diffusion-weighted data were acquired using three b-values 0, 1000, 2000 s/mm^2^ with diffusion encoding along left-right direction. As the GRAPPA reconstruction is relatively independent to the diffusion encoding directions, the evaluation results of ACS acquisition should be similar for all diffusion directions. ACS data were acquired using spin-echo SSH-EPI, conventional spin-echo segmented EPI, Slab-FLEET and Slice-FLEET respectively, and to evaluate the effects of slab/slice thickness on FLEET-ACS acquisition, four different slab thicknesses (100%, 50%, 20% and 10%) were measured for the Slab-FLEET, and two slice thicknesses (1× and 2× respect to the final imaging slice) were measured for the Slice-FLEET, resulting in 8 sets of ACS data. The total acquisition time for the ACS data and the image data was about 6 min.

#### 3.2.2 High-resolution diffusion protocol

Diffusion-weighted images were acquired with *b*=1500 s/mm^2^ and 64 diffusion directions uniformly sampled on a sphere, and eight *b*=0 images (six of these acquired with anterior–posterior phase encoding to match the high-b scan, and two acquired with opposite phase encoding for distortion correction). Other sequence parameters were the same as the ACS evaluation protocol except a longer TR of 2700 ms was used due to SAR limitation. One set of *b*=0 data was oversampled by a factor of 2 along the *k*_z_ direction for the initial estimation of slab profile required by NPEN. Two sets of ACS data were acquired using Slab-FLEET with 50% slab thickness and Slice-FLEET with 1× slice thickness, which were used to reconstruct navigator signal and imaging signal respectively. The total scan time for all diffusion-weighted data was ~35 min.

### 3.3 Reconstruction

#### 3.3.1 GRAPPA reconstruction

GRAPPA reconstructions using ACS data acquired with spin-echo SSH-EPI, conventional spin-echo segmented-EPI and Slab-FLEET followed the procedures described previously (Engstrom and Skare, 2013). In short, for each slab, a 2D GRAPPA kernel was calibrated and applied to synthesize the missing *k*-space lines for all *k*_z_ encodes and the navigator. After the GRAPPA reconstruction, motion-induced phase errors for each *k*_z_ segment were corrected using the phase of the navigator images. Finally, a 3D Fourier transform produced the image.

**Fig. 2:**
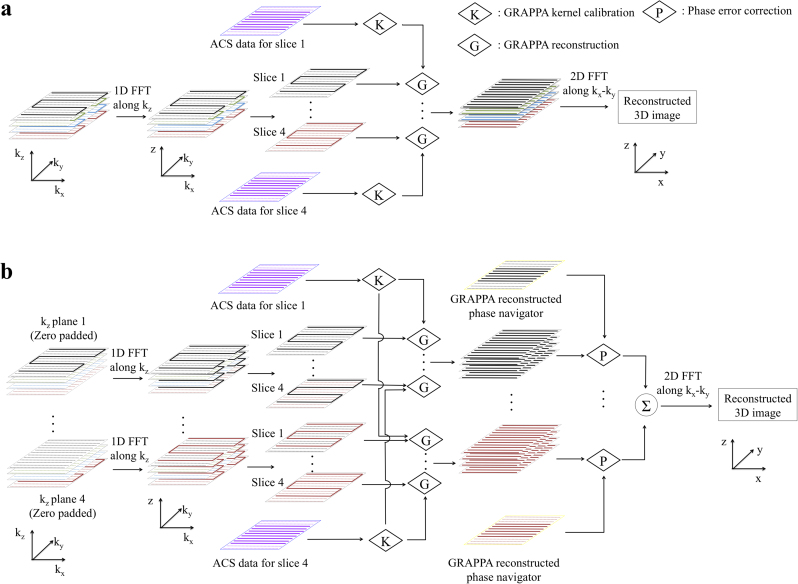
GRAPPA reconstruction and phase error correction for 3D multi-slab MRI with Slice-FLEET ACS: (a) *b*=0 data, (b) Diffusion-weighted data.

The reconstruction pipeline needs to be altered when using ACS data acquired with Slice-FLEET, which uses a GRAPPA kernel calibrated for each individual slice. In order to reconstruct the imaging data, a Fourier transform along *k*_z_ should be applied to transform from the *k*_x_−*k*_y_−*k*_z_ space to the *k*_x_−*k*_y_−*z* space before the slice-by-slice in-plane GRAPPA reconstructions (Fig. 2a). For *b*=0 data this works fine, but for diffusion-weighted images this initial *k*_z_ Fourier transform becomes problematic due to phase-inconsistencies between segments. These phase errors need to be corrected using the navigator echoes, but the navigator correction itself needs full-FOV images (i.e. images that already have been GRAPPA reconstructed). GRAPPA reconstruction therefore is a circular problem when applied to diffusion-weighted images in combination with Slice-FLEET ACS data. To solve this problem, a new scheme (Fig. 2b) was proposed by exploiting the linearity of each reconstruction stage, which meant that the contribution of each 3D *k*-space segment to each *z* plane (slice) could be calculated independently. Specifically, every individual *k*_z_ segment was first placed in an otherwise zero-filled 3D *k*-space, and then Fourier transformed along *k*_z_. This avoids the different levels of phase corruption between segments to interfere with one another, in other words: it is safe to perform a *k*_z_ Fourier transform despite the phase errors not having been corrected yet. We then applied slice-by-slice GRAPPA reconstruction, followed by phase navigator correction. Finally, the *N*_kz_ *k*-spaces were summed and Fourier transformed to generate a 3D slab image (i.e. invoking the linearity of individual stages).

For Slice-FLEET with 2× slice thickness, GRAPPA reconstruction for an image slice used kernels calibrated from the nearest FLEET slice.

#### 3.3.2 NPEN correction

The method for correcting slab boundary artefacts using NPEN has been described previously (Wu et al., 2015). In short: NPEN formulates the 3D multi-slab acquisition as a nonlinear inversion problem, where both the slab profiles and the underlying images are treated as unknowns and solved using iterative methods. The slab profile (but not the image) is constrained to be smooth in each slice plane to improve the conditioning of the problem, and a penalty term is applied to reduce slab boundary artefacts, which are assumed to be periodic along the slice direction.

Initial estimations are required for nonlinear inversion, which was previously based on the *b*=0 image (Wu et al., 2015). A potential drawback of this method is that if the *B*_0_ field is very inhomogeneous, the slab profile can be locally distorted and shifted, and as a result, the initial 3D slab profile estimated from the averaged signal within slice may not be suitable at all slice locations, which can lead to biased reconstructions. At 3T, this effect was not seen due to relatively homogeneous *B*_0_ and high time-bandwidth product of RF pulses. However, this does become problematic at 7T due to increased *B*_0_ inhomogeneity and the use of lower time-bandwidth product pulses due to SAR limitations.

To mitigate this problem, slab profile initialisation was modified to include a rough estimation of in-plane profile variation caused by *B*_0_ inhomogeneity. First, a Bloch simulation of the spin-echo sequence used in this work was performed with a range of off-resonance frequencies to generate a lookup table for MRI signals with voxel location and off-resonance frequency as entries, which only needs to be done once if the sequence parameters are not changed. A field map was estimated from the eight *b*=0 images with both normal and reversed phase using FSL's TOPUP tool (Andersson et al., 2003). With the field map and the lookup table, the distorted slab profile could be produced within seconds. Then a 2D weighting map was generated for each slice by normalizing the simulated profile with the averaged magnitude within slice. After that, the initially estimated 3D slab profile was multiplied with the weighting maps to formulate the final slab profile estimation. It should be noted that, as with any field map based correction method, with substantial subject motion, the slab profile estimation would become inaccurate if the field map was not updated. To alleviate this problem, the multiple *b*=0 image pairs (with normal and reversed phase) could be arranged at different stages of the scan to generate field maps at different time points.

### 3.4 Post-processing

Image distortions induced by susceptibility and eddy currents were estimated and corrected using TOPUP and EDDY (Andersson and Sotiropoulos, 2016). We then fit the tensor model using the DTIFIT tool (Smith et al., 2004) and multiple fibre populations within a single voxel using BedpostX (Behrens et al., 2007). White matter tractography was performed in the diffusion space using FSL's probabilistic tracking tool (Behrens et al., 2003, Behrens et al., 2007). Two major association fibre tracts (cingulum bundle and superior longitudinal fasciculus, SLF) were investigated with seed masks drawn on a single coronal slice in the diffusion space (Supplementary Fig. S1), no target or stopping masks were used. An exclusion mask at mid-brain was applied to avoid cross-hemispheric tracts. Step length of 0.2 mm and 5000 streamlines were used in the tractography with no curvature threshold. To evaluate the quality of tractography, we compared these results to high-resolution post-mortem data presented in previous work (Miller et al., 2012).

## 4 Results

### 4.1 Comparison of different ACS acquisitions

**Fig. 3:**
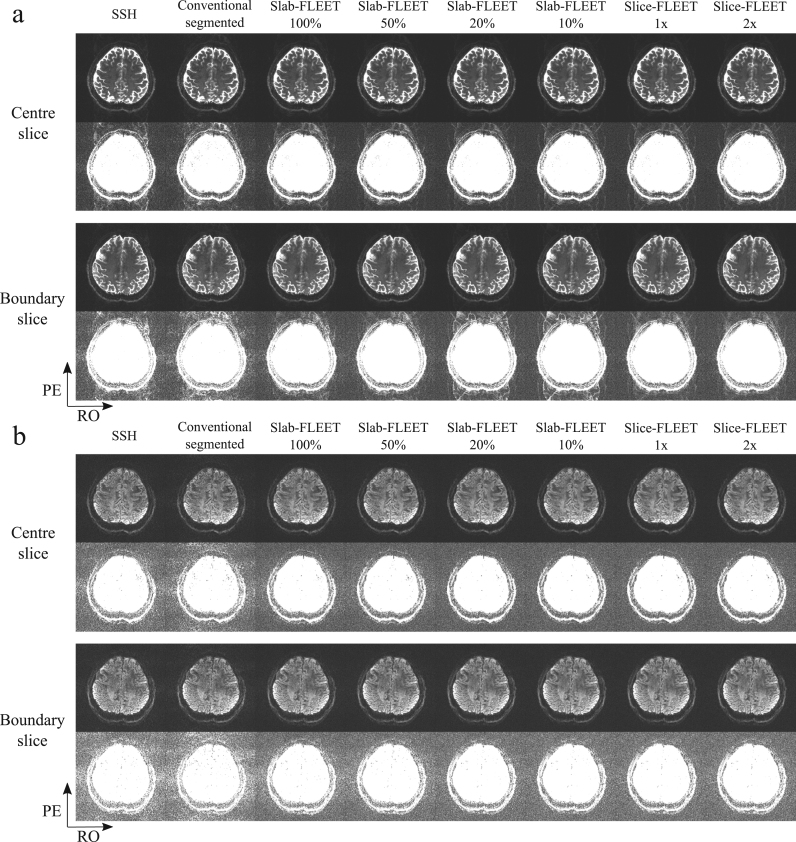
Comparison of GRAPPA reconstructions using different ACS acquisition methods. (a) *b*=0 images. (b) Diffusion-weighted images acquired with *b*=1000 s/mm^2^. In each figure, the top two rows show a slice at slab centre and the bottom two rows show a slice at slab boundary. The phase encoding (PE) direction is along anterior–posterior.

**Fig. 4:**
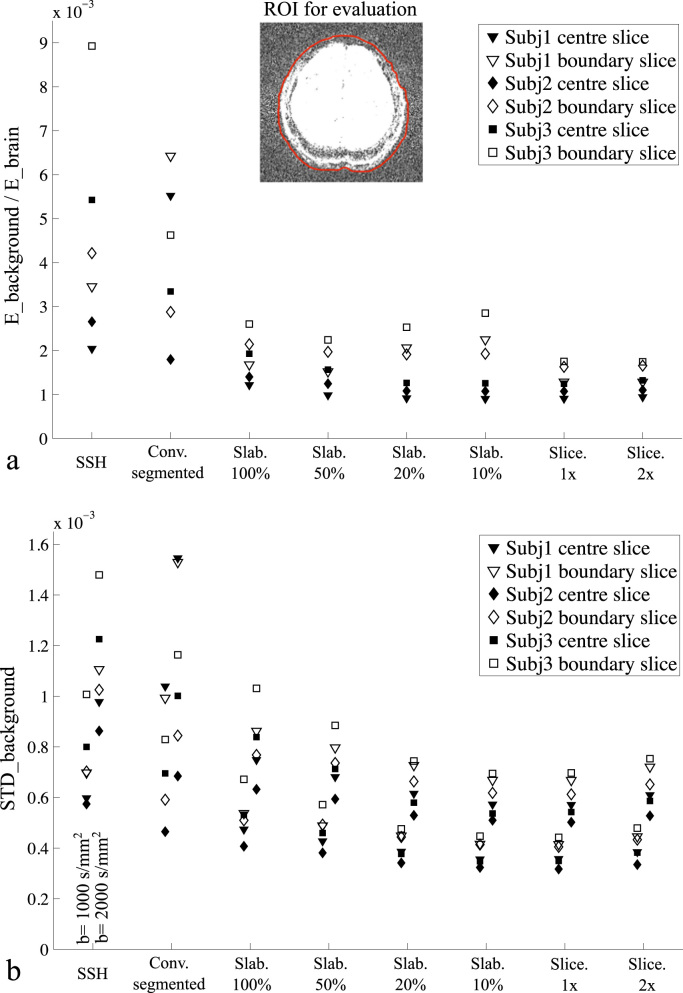
Quantitative comparison of different ACS acquisition methods for three subjects. (a) Ratio between signal energy in the background region (E_background) and signal energy in the brain region (E_brain) for *b*=0 data, used as a measurement of residual aliasing. (b) Standard deviation of background signal for *b*=1000 s/mm^2^ and *b*=2000 s/mm^2^ data, used as a measurement of noise level. A region of interest (ROI) for the brain of one subject is shown in (a) and the background region is defined accordingly, which are used to evaluate ghosting artefacts and noise level.

Fig. 3 compares GRAPPA reconstructions for *b*=0 data (Fig. 3a) and diffusion-weighted data (Fig. 3b, *b*=1000 s/mm^2^) with ACS data acquired using different methods. ACS data acquired with SSH-EPI suffers from strong residual aliasing and noise amplification, likely due to the mismatched distortions between ACS data and imaging data. ACS data acquired using segmented EPI match distortion, but nevertheless display strong residual artefacts and amplified noise, which is likely due to subject motion and/or respiration during the long ACS acquisition time. All FLEET-ACS methods are observed to have reduced artefact. The choice of slab thickness in Slab-FLEET is not trivial: a thin slab thickness (e.g. 20%, 10%) allows good GRAPPA reconstruction at slab centre, but fails to provide good results at slab boundaries, presumably because these regions are not considered during GRAPPA kernel calibration. This is avoided when a thick slab thickness (e.g. 50%, 100%) is used, which however can lead to strong noise amplification, especially in the diffusion-weighted images (e.g. the noise level of the GRAPPA reconstruction using Slab-FLEET (50%) ACS is ~20% higher than that using Slice-FLEET (1×), see Fig. 4b). This might be caused by the strong intra-voxel dephasing effects within thick slabs and inaccurate estimation of local coil sensitivity. In contrast, Slice-FLEET performs well in both scenarios with 1× and 2× slice thicknesses showing good reconstructions.

A quantitative comparison of different ACS acquisition methods for three subjects is shown in Fig. 4. For the *b*=0 data, the ratio between signal energy in the background region and signal energy in the brain region was calculated and used as a measurement of residual ghosting artefacts (Fig. 4a). For the diffusion-weighted data, the standard deviation of background signal was used as a measurement of noise level (Fig. 4b). It can be seen that FLEET-ACS methods enable more robust GRAPPA reconstruction for all subjects compared to SSH-EPI and conventional segmented EPI. Among all FLEET-ACS schemes, Slice-FLEET exhibits superior performance with low aliasing artefacts and low noise amplification for both centre slice and boundary slice.

**Fig. 5:**
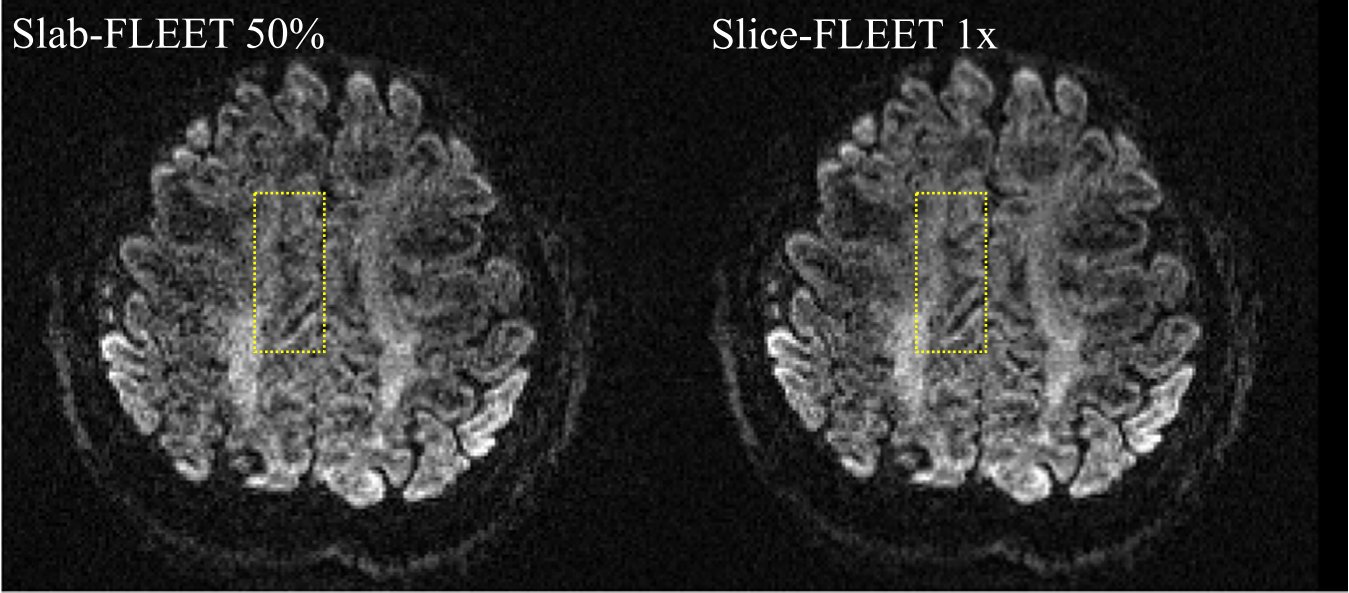
Comparison of GRAPPA reconstruction using ACS data acquired with Slab-FLEET (50% slab thickness) and Slice-FLEET (1× slice thickness). *b*=2000 s/mm^2^. The Slice-FLEET method demonstrates superior SNR and better preservation of detailed brain structures than Slab-FLEET (yellow rectangle). (For interpretation of the references to color in this figure legend, the reader is referred to the web version of this article.)

Fig. 5 shows a more specific comparison between Slab-FLEET and Slice-FLEET. A slice at slab centre with a b-value of 2000 s/mm^2^ is reconstructed using ACS data acquired with Slab-FLEET (50% slab thickness) and Slice-FLEET (1× slice thickness) separately. As shown in the results, Slice-FLEET ACS enables superior SNR in GRAPPA reconstruction and better preservation of detailed brain structures than Slab-FLEET method, for example, the diffusion-weighted contrast where white matter fibres enter a gyrus on the medial surface of the right hemisphere is clearer in the Slice-FLEET result (yellow rectangle).

Based on the results of the comparison, the diffusion-weighted data acquired with the high-resolution diffusion protocol were reconstructed using Slice-FLEET ACS data.

### 4.2 Slab boundary artefacts correction

**Fig. 6:**
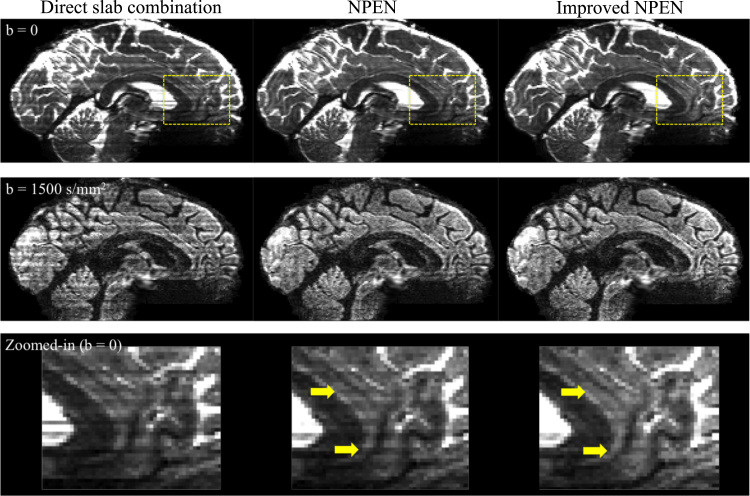
Correction of slab boundary artefacts using direct slab combination, NPEN and improved NPEN. Slab boundary artefacts are effectively reduced using NPEN correction, although there are still some visible residual artefacts in the areas with strong B_0_ inhomogeneity (yellow arrows in the zoomed-in version). Improved NPEN further reduces these artefacts. Note that due to the low intensity of the diffusion-weighted images in the inferior frontal region, the differences between the improved NPEN and previously proposed NPEN are less clearly visible. (For interpretation of the references to color in this figure legend, the reader is referred to the web version of this article.)

Fig. 6 shows the results of slab boundary artefacts correction. Three methods are compared here: direct slab combination (the first and the last slices of each slab are discarded and the remaining slices from all slabs are concatenated), NPEN and improved NPEN. The direct slab combination retains strong artefacts at slab boundaries. NPEN effectively reduces the artefact level. However, NPEN correction still contains some residual artefacts in the region of strong B0 inhomogeneity (yellow arrows in the zoomed-in image). With improved NPEN, these residual artefacts are further reduced (zoomed-in image).

### 4.3 Diffusion tensor results

**Fig. 7:**
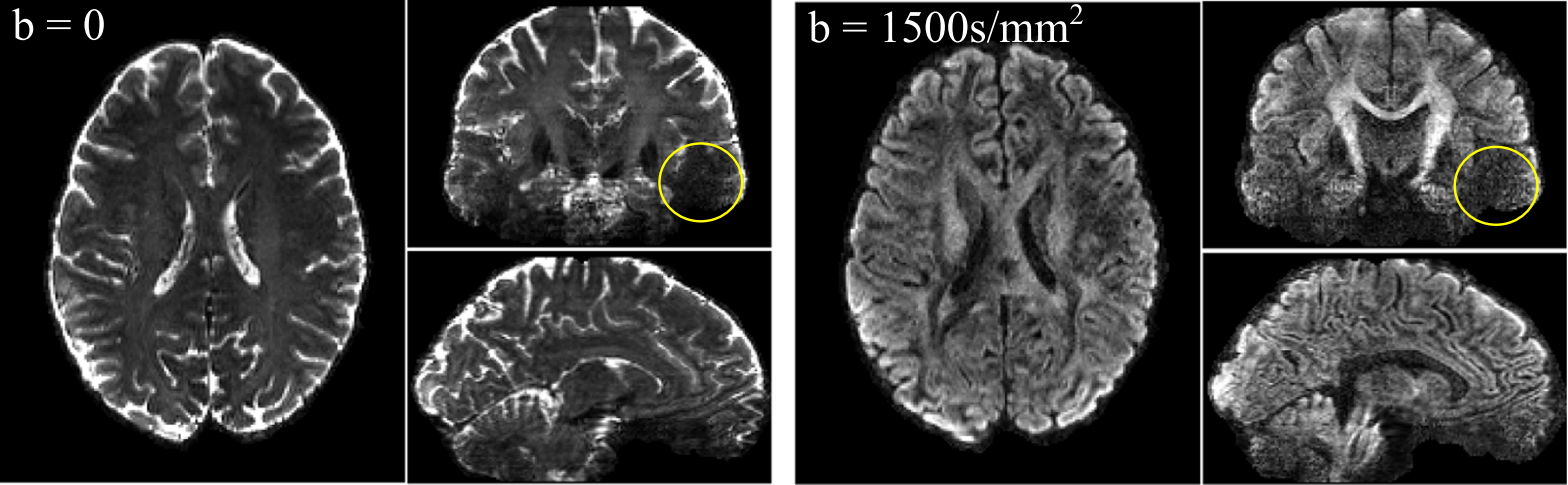
High-resolution data acquired at 7T using the high-resolution diffusion protocol. The yellow circles at temporal lobes indicate signal drop caused by the severe *B*_1_^+^ inhomogeneity at 7T. (For interpretation of the references to color in this figure legend, the reader is referred to the web version of this article.)

Fig. 7 shows high-resolution (~1 mm isotropic) *b*=0 and diffusion-weighted images (*b*=1500 s/mm^2^) acquired at 7T with a scan time of 27 s per volume. The images show high SNR, high contrast and good anatomical detail thanks to the high resolution. It should be noted that the low signal intensity in the temporal lobes (yellow circles) is due to the inhomogeneity of transmission field (B_1_^+^) at 7T.

**Fig. 8:**
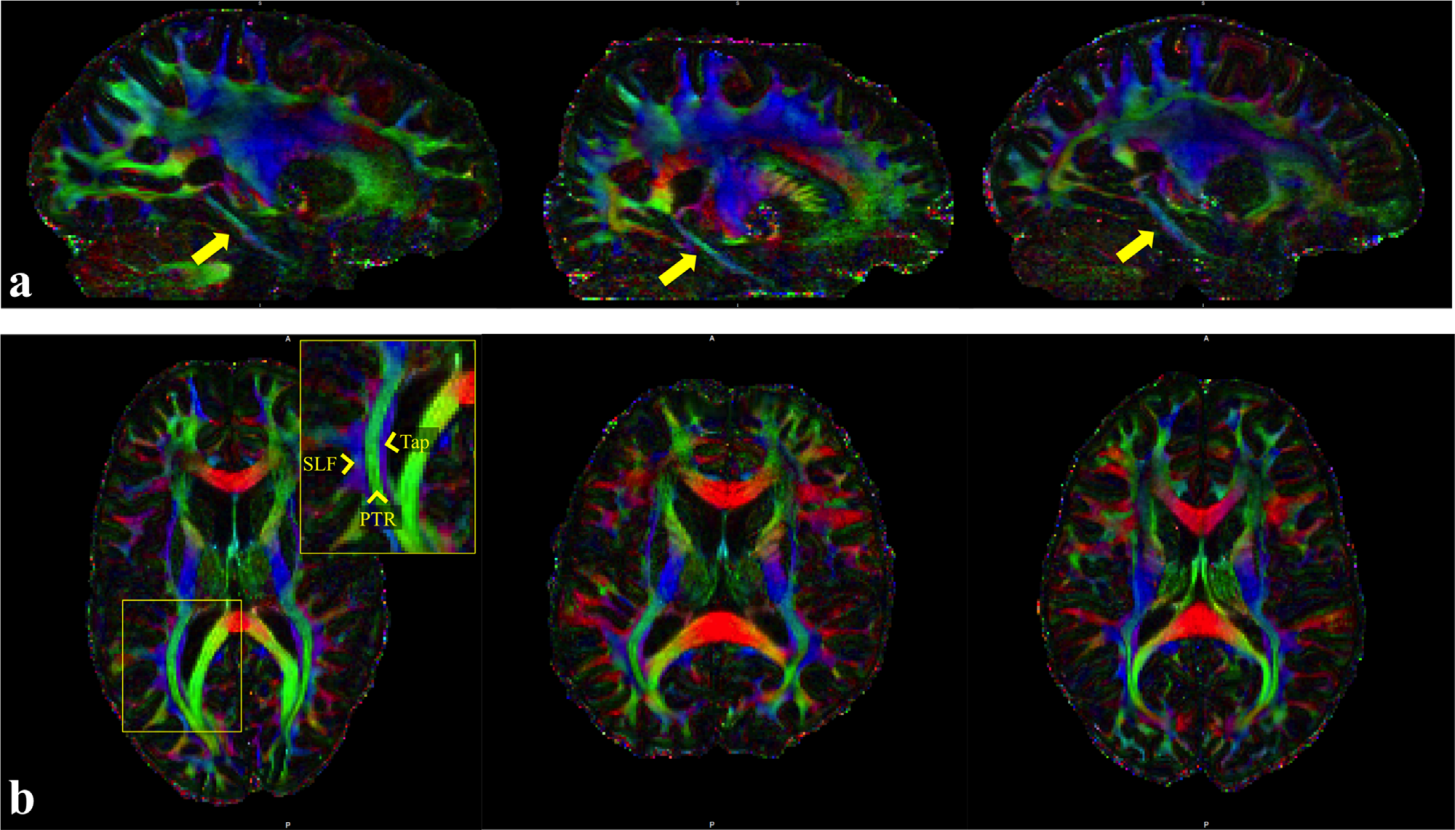
Colour-coded maps of the principle eigenvectors for the high resolution DTI data (red, right–left; green, anterior–posterior; blue, superior–inferior). The high spatial resolution enables (a) clear detection of thin fibre tracts such as the cingulum bundle descending into the temporal lobe (yellow arrows) and (b) crisp disambiguation of some fibres which can be difficult to separate using low resolution diffusion MRI (superior longitudinal fasciculus, SLF, posterior thalamic radiation, PTR, the tapetum of the corpus callosum, Tap). (For interpretation of the references to color in this figure legend, the reader is referred to the web version of this article.)

Fig. 8 shows colour-coded maps of the principle eigenvectors from three subjects. High data quality is evident, for example, in the cingulum bundle as it descends into the temporal lobe toward hippocampal projections (Fig. 8a, yellow arrows), a thin tract but here it is several voxels thick. Fig. 8b shows the white matter tracts near the posterior horn of the lateral ventricles: the superior longitudinal fasciculus, posterior thalamic radiations and tapetum are nicely distinguished in the high-resolution in vivo DTI data.

**Fig. 9:**
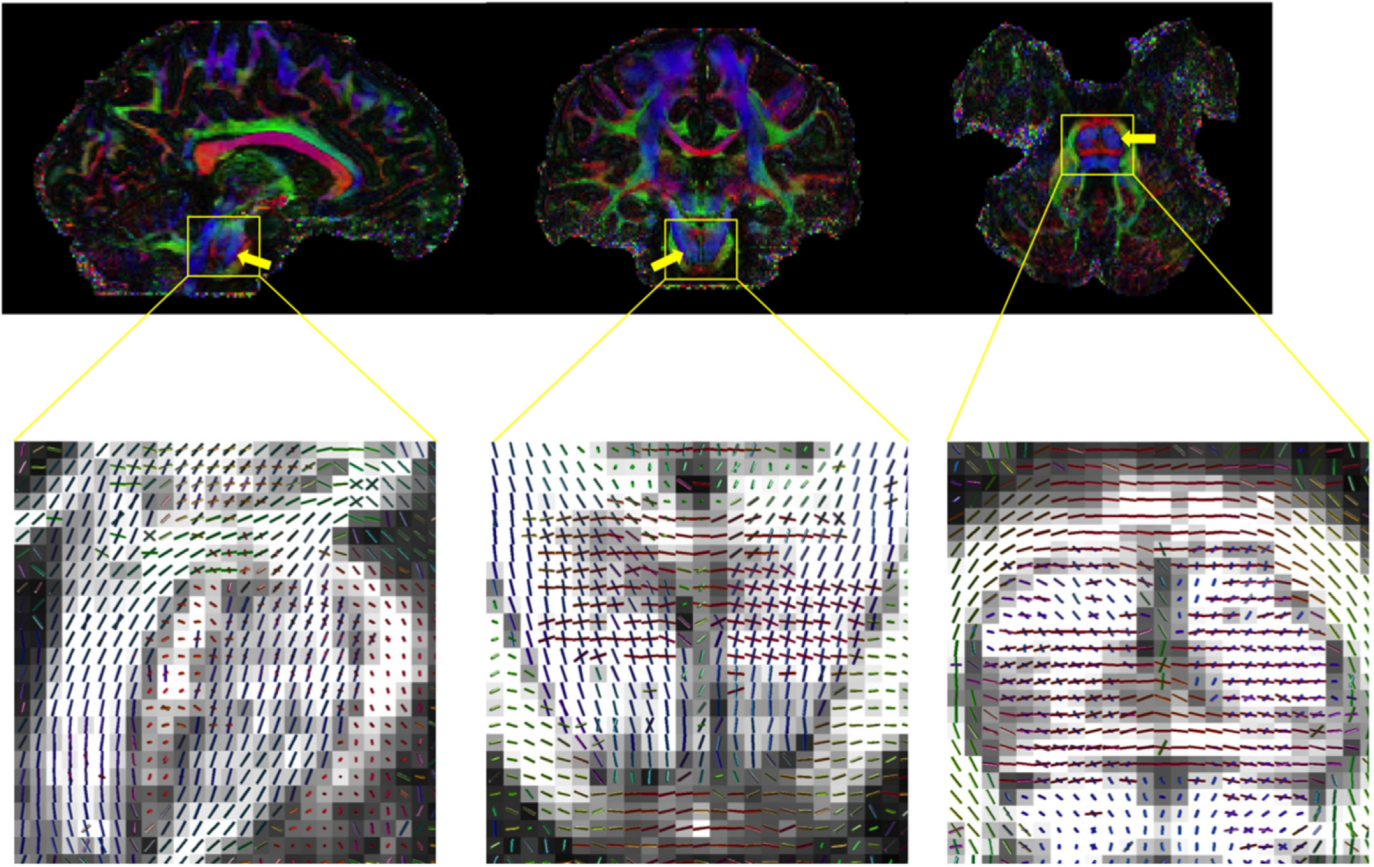
Complex fibre architecture in the pons is robustly detected in our high-resolution diffusion data. Top row: colour-coded maps of principle eigenvectors (red, right–left; green, anterior–posterior; blue, superior–inferior). Bottom row: Zoomed region of the pons showing multiple fibre populations estimated using the ball and sticks model, demonstrating the ability of the high-resolution data to resolve crossing fibres. In particular, tensor fits in the regions indicated by the arrows in the top row are dominated by tracts running superior–inferior, but additional right–left fibre populations are accurately captured with the crossing fibre models. (For interpretation of the references to color in this figure legend, the reader is referred to the web version of this article.)

Fig. 9 shows the colour maps and fibre reconstruction of the pons of one subject, where the corticospinal tract (running superior–inferior, blue) interdigitate with the decussation of the pons (crossing right–left, red). The top row shows colour maps from a single fibre reconstruction, in which the corticospinal tracts dominate (blue, indicated by the arrows). Nevertheless, the multi-fibre reconstructions on the bottom row are able to robustly estimate both fibre populations within a voxel, demonstrating high contrast and high SNR of the data.

**Fig. 10:**
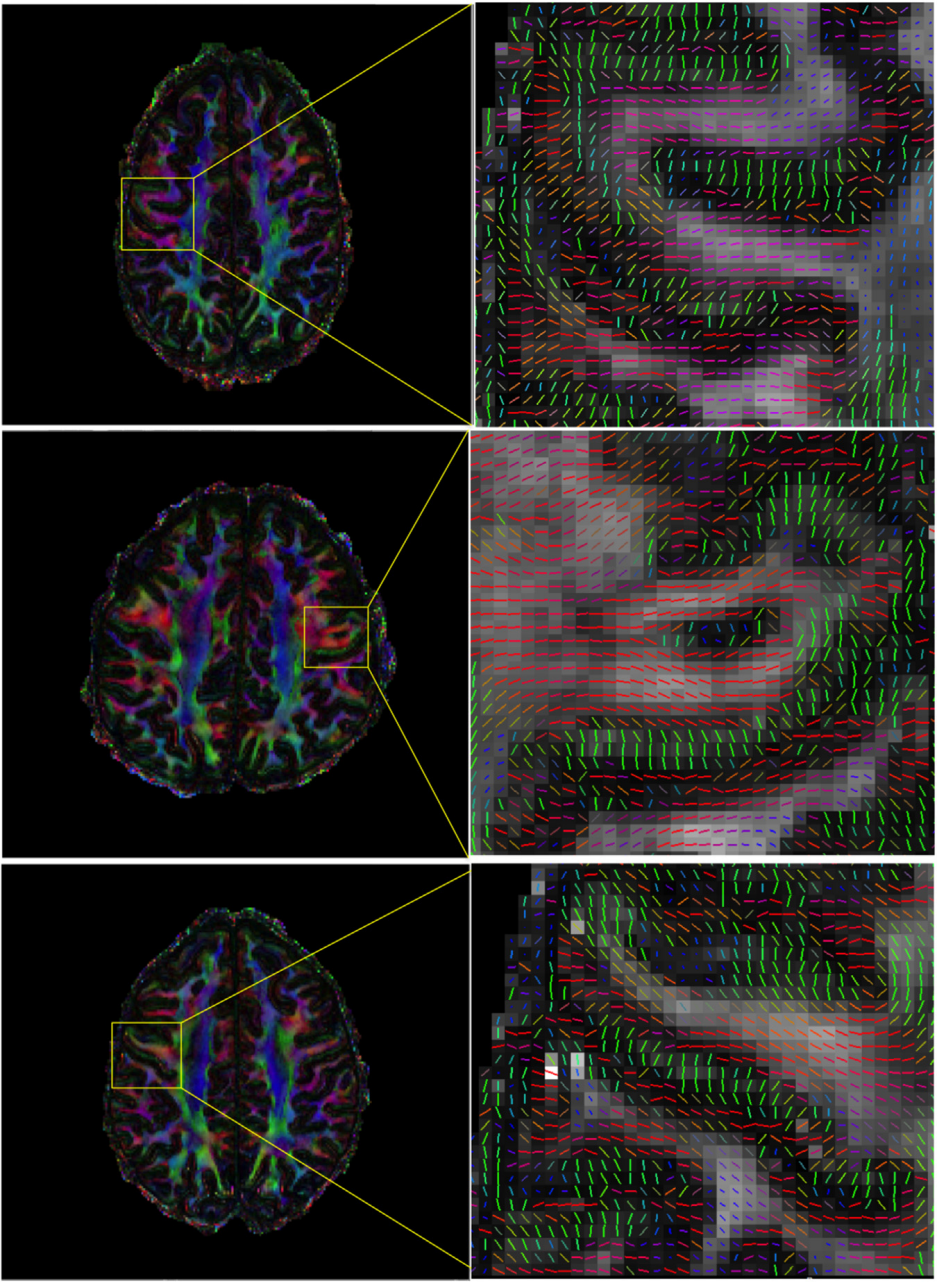
Depiction of cortical anisotropy using high-resolution DTI data (red, right–left; green, anterior–posterior; blue, superior–inferior).

Fig. 10 demonstrates high-resolution depiction of cortical anisotropy, focusing on the central sulcus. For all three subjects, the high-resolution data consistently supports visualisation of fibre architecture within gyri. Here, the precentral gyrus is depicted in an axial plane just above the superior-most arch of the cingulum bundle. In all subjects, fibres can be seen entering the gyrus, turning slightly and entering cortex, with particularly clear delineation of radial orientation on the anterior bank of the central sulcus (i.e. primary motor cortex) and tangential orientation on the posterior bank of the central sulcus (i.e. primary somatosensory), consistent with previous reports (McNab et al., 2013).

### 4.4 Tractography results

**Fig. 11:**
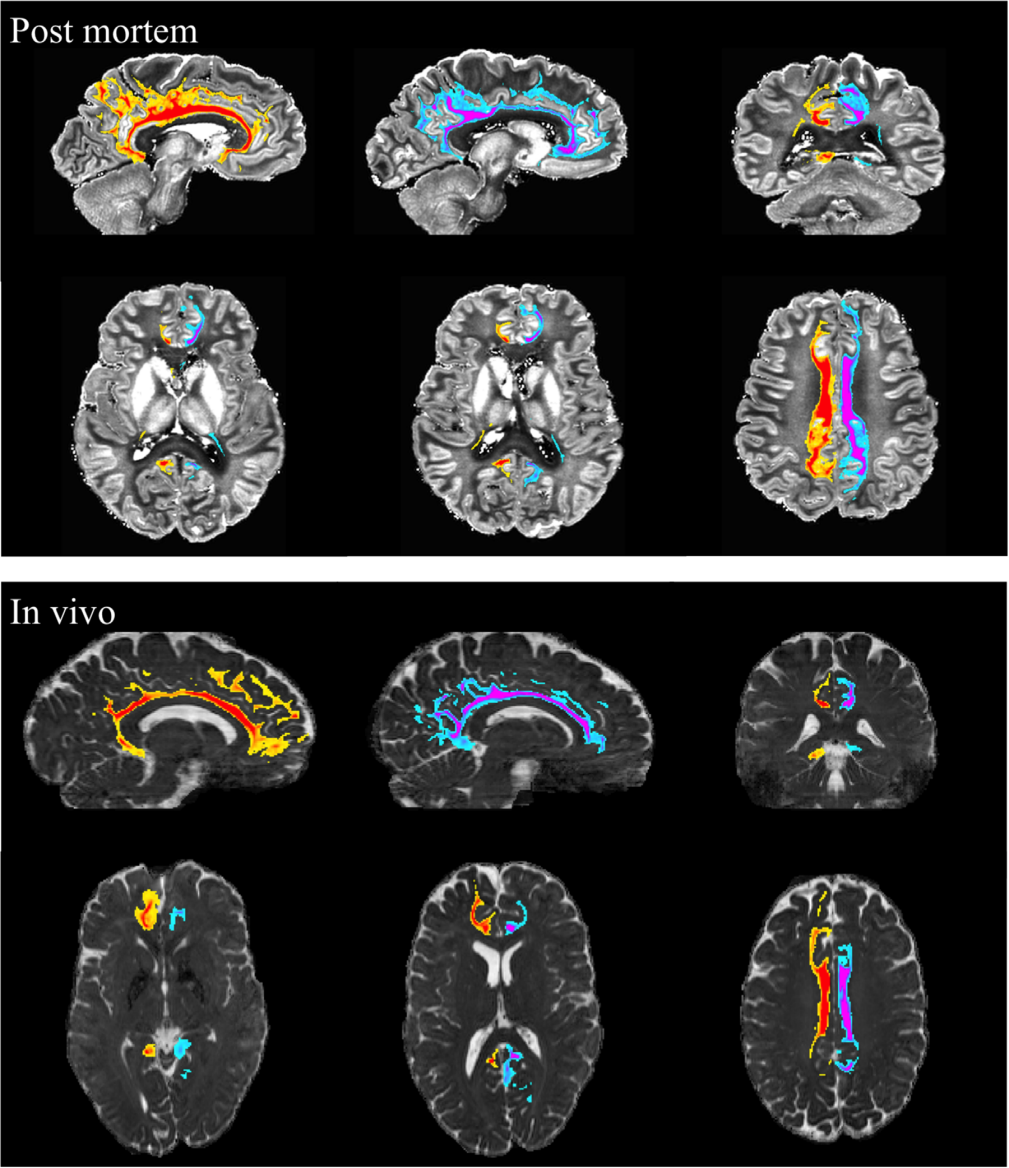
Tractography results obtained from the left (blue–magenta) and right (yellow–red) seed regions in the cingulum bundle. Top row: for reference, post-mortem data acquired with 0.94 mm^3^ isotropic resolution using 3D diffusion weighted steady state free precession at 3T. Bottom row: in-vivo data acquired with 1.03 mm^3^ isotropic resolution using spin-echo 3D multi-slab EPI at 7T. All results are rendered with a threshold of 100 streamlines and overlaid on mean diffusivity maps. (For interpretation of the references to color in this figure legend, the reader is referred to the web version of this article.)

**Fig. 12:**
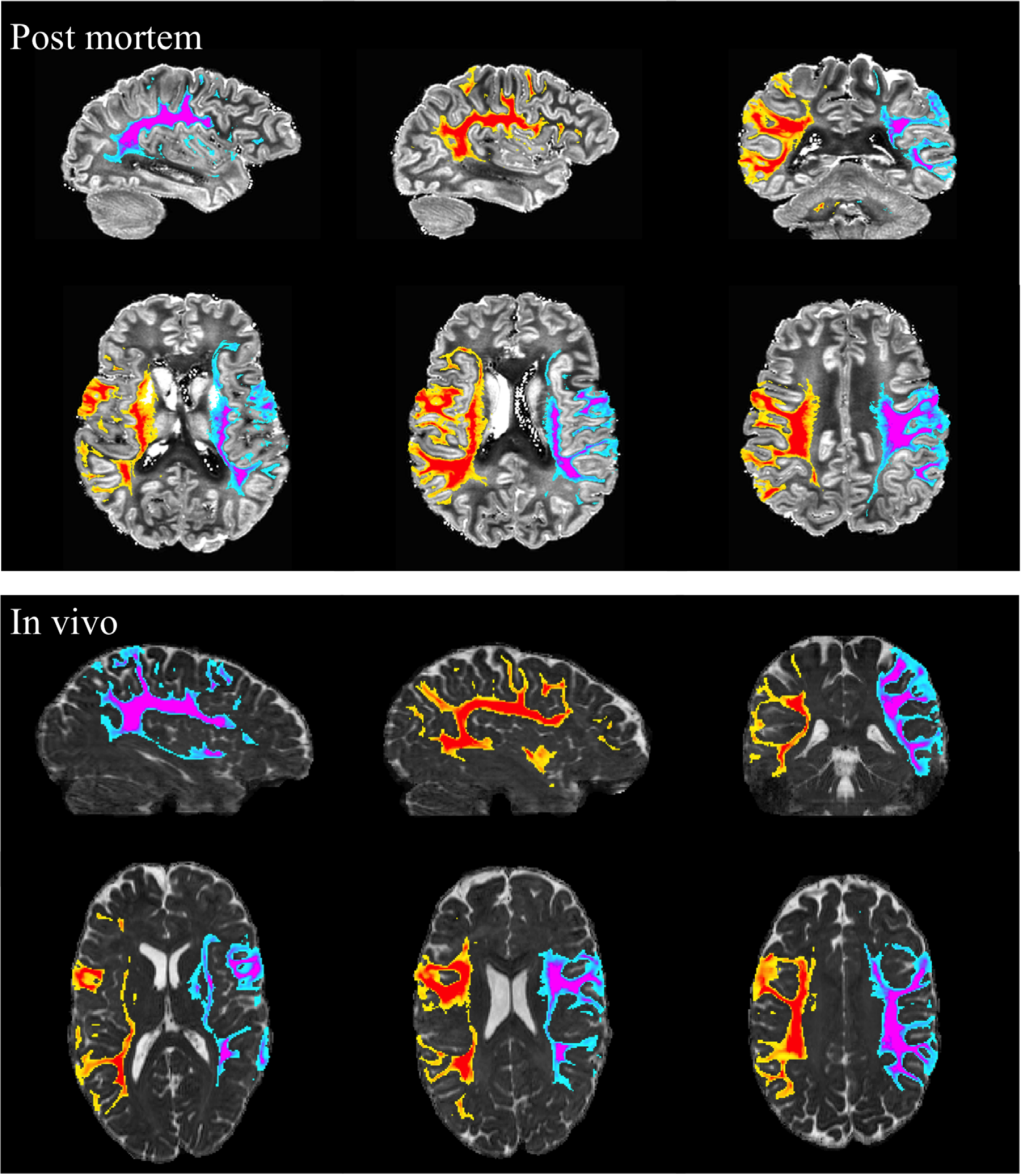
Tractography results obtained from the left (blue–magenta) and right (yellow–red) seed regions in SLF bundle for post-mortem data (top row) and in-vivo data (bottom row). All results are rendered with a threshold of 100 streamlines and overlaid on mean diffusivity maps. (For interpretation of the references to color in this figure legend, the reader is referred to the web version of this article.)

Tractography of the cingulum bundle and SLF are shown in Fig. 11 and Fig. 12, respectively. For comparison, tractography from post-mortem data in a previous study are also shown (Miller et al., 2012) (6 h scan at 3T using a diffusion-weighted steady state free precession sequence (Mcnab et al., 2009)) and used as gold standard. Even with these relatively simple tractography analyses (using a single seed mask, a mid-line exclusion mask and no waypoint masks), the in vivo tractography captures essentially the entire extent of the cingulum bundle, including temporal and frontal lobe tracts and cortical projections into cingulate cortex along its entire extent. The SLF tractography on the in vivo data also demonstrates consistency with the post-mortem data, including tracks into the pars triangularis and pars opercularis of the inferior frontal gyrus, and tracks into the supramarginal gyrus and angular gyrus in the parietal lobe.

## 5 Discussions

We have demonstrated the acquisition of excellent quality, high-resolution (1 mm isotropic) diffusion imaging through the combination of 7T and 3D multi-slab imaging, the latter enabling TRs in the optimal range with respect to SNR efficiency. While the multi-slab approach considerably reduces the coupling between coverage and TR, the shortest possible TR for 3D multi-slab acquisition is to an extent limited by the FOV along the slab direction and the slab thickness. Thin slabs are preferred as they allow better phase-error correction with 2D navigators, while large FOV is necessary when full brain coverage is required. As a result, the shortest TR we could use in practical is slightly longer than the TR associated with optimal SNR efficiency. Nevertheless, the TR used in this work (2.6/2.7 s) provides a considerably higher SNR efficiency than the typical TR (>7 s) that is commonly used for 2D diffusion MRI at 7T. The use of thin slabs might be less robust to truncation artefacts due to the small number of *k*_z_ encodes, but this is not clearly visible in our results. The lengthening of *T*_1_ at 7T is a disadvantage for SNR, but its effect is not substantial in this application (7T vs. 3T SNR ratio is decreased by ~7% due to the increase in *T*_1_ with the TRs used in this work) compared to the signal gains from the increase in field strength.

Another advantage of 3D multi-slab acquisition is its intrinsically higher SNR for each diffusion volume, which is beneficial for reducing the bias due to the non-Gaussian noise distribution, particularly in high-resolution diffusion MRI scan (Fürsich et al., 2015). Compared to simultaneous multi-slice imaging, the primary disadvantage of 3D multi-slab imaging is the increased scan time per volume, which reduces the number of diffusion directions that can be achieved using the same scan time. The scan protocol used in this work allows more than 50 diffusion directions in a 30 min scan, which should be sufficient for many in vivo diffusion MRI studies. One promising further improvement to this sequence would be the acquisition of multiple non-contiguous slabs simultaneously (i.e. the 3D extension of simultaneous multi-slice imaging), which would both reduce the excitation TR and the total scan time (Frost et al., 2013, Schulz et al., 2016).

It should be noted that the SAR predicted by the system is affected by some subject-specific factors, such as subject's weight and coil load. In the ACS evaluation protocol, we used RF pulses with a high time-bandwidth product to achieve a good slab profile, which resulted in a high SAR value when scanning the three subjects involved in this experiment. Three different subjects were subsequently scanned with the high-resolution diffusion MRI protocol, at which point the SAR limits were exceeded for one subject. We decided to increase the TR slightly (100 ms) to satisfy the SAR constraint without changing other sequence parameters.

Due to the severe B_0_ inhomogeneity and short tissue *T*_2_^*^ at 7T, in-plane parallel acceleration is critical for SSH-EPI. The choice of acceleration factor is a tradeoff between the reduction of image distortion and blurring versus the amplification of noise. In this work, we used an in-plane GRAPPA factor of 3, which provides substantial reduction of image distortion and sufficient image SNR (Vu et al., 2015). It should be noted that, due to the *T*_2_^*^ decay during the EPI echo train, the *k*_y_ phase encoding direction exhibits the largest blurring effect. With the acquisition protocol used in this work, the additional blurring in WM induced by the *T*_2_^*^ (~25 ms (Yacoub et al., 2001)) decay is ~20% (only in the in-plane phase-encoding direction), measured by the full-width at half maximum (FWHM) of the point spread function (Haacke et al., 1999).

In accordance with previous work (Polimeni et al., 2016), both Slab-FLEET ACS and Slice-FLEET ACS demonstrate greatly improved image quality compared with the conventional methods (SSH-EPI, conventional segmented EPI), including reduced residual artefacts and better-preserved SNR, which are crucial for achieving high-quality diffusion MRI data. The difference in contrast between the gradient-echo FLEET ACS data and spin-echo imaging data does not appear to introduce substantial effects in the reconstruction, consistent with previous reports that GRAPPA is relatively insensitive to contrast differences (Griswold et al., 2006). Slice-FLEET acquires ACS for each slice, requiring considerably longer scan time (~58 s for 114 slices using 5 dummy scans) than Slab-FLEET (~7 s for 14 slabs using 5 dummy scans) but better captures local coil sensitivity and reduces intra-voxel dephasing. This time penalty can be halved by using doubled slice thickness, without any visible differences in the GRAPPA reconstruction results (Fig. 3). Considering also that only one ACS acquisition is required for all directions, Slice-FLEET has only a very minimal impact on the total scan time while providing the best reconstructions. The navigator signal is acquired without *k*_z_ phase encoding, so the Slab-FLEET ACS is an apparent option for GRAPPA reconstruction and was used in this work, which added ~7 s on the total scan time. Nevertheless, the need for Slab-FLEET ACS could be also avoided by using slab-wise averaged Slice-FLEET ACS data, which should be able to provide similar results. Another possible FLEET-ACS scheme for multi-slab acquisition is to acquire 3D ACS slabs with full *k*_z_ encodes. However, this scheme is expected to be very sensitive to subject motion and respiration due to the long interval between the acquisitions of the first and the last segments (i.e. precisely the reason why FLEET-ACS was proposed for 2D EPI), we therefore did not consider it in this work.

It is worth noting that in this work the GRAPPA reconstruction was applied to each *k*_z_ plane individually instead of using a true 3D GRAPPA reconstruction, which might further improve the reconstruction. Further studies will be needed to investigate the performance of 3D GRAPPA reconstruction on the 3D multi-slab data, which is beyond the scope of this paper.

Slab boundary artefacts are one major challenge with 3D multi-slab sequences. The recently proposed NPEN method has demonstrated its ability to reduce slab boundary artefacts effectively and robustly at 3T (Wu et al., 2015). However, at 7T, this method has to contend with increased *B*_0_ inhomogeneity and reduced RF bandwidths due to SAR limitations, which distort and shift the slab profile. In this work, we modified the initialisation of the slab profile by incorporating B_0_ effects, which could provide a more accurate start point for the iterative reconstruction. However, even with this modification, there are still some residual boundary artefacts visible in the region of strong B_0_ inhomogeneity, presumably because the slab shifting and distortion interfere with the NPEN assumption that artefacts are periodic along the slice dimension. A possible solution to this problem is to estimate a warping matrix based on the field map and include it in the reconstruction to restore the periodicity of the artefacts, such that it satisfies the assumption of NPEN. However, due to the slab aliasing, a single warping matrix cannot correctly restore both the underlying signal and the aliasing signal from one voxel, which might lead to incorrect reconstruction. Further investigation is needed to improve the NPEN correction under strong B_0_ inhomogeneity. Nevertheless, despite these residual artefacts, we are able to reconstruct high quality visualisations of fibre architecture from the data.

Subject motion is a major challenge for multi-shot diffusion MRI. In this work, a navigator based nonlinear correction method was applied to correct the motion induced phase errors. In general, the acquisition of 3D navigator is infeasible due to the long time required to traverse 3D k-space, although some groups have considered methods for batching 3D navigators across multiple repetitions (McNab et al., 2010). In the current work, a 2D navigator was used in the correction, assuming that the phase error along the slice direction is constant if a thin slab is acquired (Engstrom and Skare, 2013). To the extent that this assumption does not hold, there could be residual artefacts in the data, which might alter the quantification of diffusion anisotropy as they are dependent on diffusion directions. Further studies are needed to evaluate the effect of these residual artefacts. It should be noted that if subject motion is very strong, neither 2D nor 3D nonlinear corrections could address the phase errors. In this case, methods like real-time image rejection and re-acquisition could be helpful (Porter and Heidemann, 2009).

It is worth noting that, the cingulum bundle tracts from the in vivo data end earlier in the forebrain (Fig. 11), which is mainly due to the low signal level in that region caused by *B*_1_^+^ inhomogeneity and could be improved with parallel transmission or dielectric pads (Vu et al., 2015).

## 6 Conclusions

Improving the resolution of diffusion MRI has been a topic of interest recently due to its ability to provide a more accurate depiction of complex fibre architectures. However, the reduced SNR at high resolution presents a substantial challenge to diffusion MRI, severely degrading the quality of high-resolution data. In this work, we utilize two powerful tools, 7T and 3D multi-slab acquisitions, to improve the SNR of diffusion MRI. Robust imaging required modification of two existing techniques: FLEET-ACS for improved parallel imaging and NPEN correction of slab boundary artefacts. Two FLEET-ACS schemes for use with 3D multi-slab acquisition, Slab-FLEET and Slice-FLEET, were compared, with Slice-FLEET demonstrating better reconstruction quality. A new reconstruction scheme was also proposed for Slice-FLEET ACS data to make it compatible with navigator correction of phase errors. Results with ~1 mm isotropic resolution demonstrate very high quality data, revealing fine anatomical features and supporting detailed delineation of white matter tracts using tractography.

## Appendix A Supplementary material

Supplementary material Supporting FIG. S1. Seed masks used in the tractography for cingulum bundle (Fig. 11) and SLF (Fig. 12).
